# Examining the association between group context effects and individual outcomes in an interdisciplinary group-based treatment for chronic pain based on acceptance and commitment therapy

**DOI:** 10.1177/20494637211073012

**Published:** 2022-03-04

**Authors:** Helen R Gilpin, Soravis Ratanachatchuchai, David Novelli, Lance M McCracken, Whitney Scott

**Affiliations:** 1 INPUT Pain Management Unit, 8945 Guy’s and St Thomas’ NHS Foundation Trust, London, UK; 2 Health Psychology Section, Institute of Psychiatry, Psychology, and Neuroscience, 4616 King’s College London, London, UK; 3 Department of Psychology, Uppsala University, Uppsala, Sweden

**Keywords:** chronic pain, group context, acceptance and commitment therapy, pain management, group therapy

## Abstract

**Background:**

Although cognitive-behavioural treatments for chronic pain are delivered in groups, there is little research investigating group effects in these treatments.

**Purpose:**

The aim of this study was to investigate associations between group composition variables at the start of treatment and individual outcomes following intensive interdisciplinary treatment for pain based on Acceptance and Commitment Therapy.

**Methods:**

This was a secondary analysis of routinely collected observational data. Five-hundred and sixteen patients completed a standard set of demographic, pain-related and psychosocial measures at pre- and post-treatment. Intracluster correlations (ICCs) were computed to examine the clustering of outcomes within groups and multilevel models explored the association between group composition variables and individual level outcomes.

**Results:**

The ICCs for pain intensity (0.11) and interference (0.09) suggested that multilevel models were warranted for these outcomes, while a multilevel model for post-treatment depression (ICC = 0.04) was not warranted. Group percentage of participants receiving disability benefits and group mean pain intensity at pre-treatment were significantly positively associated with individual level pain intensity at post-treatment, controlling for pre-treatment individual level pain intensity. Group mean pain intensity at pre-treatment was the only group variable that significantly predicted post-treatment pain interference at the individual level. Psychosocial group composition variables were not significantly associated with individual level outcomes.

**Conclusion:**

Given the limited predictive utility of group composition variables in the current study, future research should undertake direct assessment of group level therapeutic and countertherapeutic processes to advance understanding of who benefits from group treatments for pain and how. As the variance in outcomes accounted for by group clustering was relatively small and significant within groups variance remained, research is also needed to further understand individual level factors that influence cognitive-behavioural treatment outcomes for pain.

## Introduction

Chronic pain is associated with disability, mental health difficulties and reduced quality of life and has substantial economic costs.^1–3^ Meta-analyses of randomised-controlled trials (RCTs) support the effectiveness of pain management treatments based on cognitive-behavioural therapy (CBT).^4^ The evidence for contextual forms of CBT for chronic pain, such as Acceptance and Commitment Therapy (ACT) and mindfulness-based approaches, is also growing.^5–7^ In the United Kingdom, interdisciplinary pain management treatments based on cognitive-behavioural approaches are often delivered in a group format.^8^ Despite evidence that treatments like these are effective on average, there remains a significant proportion of patients who do not benefit.^9,10^ Development of more effective cognitive-behavioural treatments will require a better understanding of the patients most likely to benefit and how.^10–12^

Numerous studies have sought to understand predictors and moderators of outcome in cognitive-behavioural pain management treatments. In general, the findings are limited and inconclusive.^11,13–15^ One reason may be that research tends to ignore the role of complex group factors. Although most studies have investigated predictors of outcome on an individual basis, participants in group-based pain treatments are embedded within a group system that brings its own unique contextual effects. Within this context, it is plausible that experiences and behaviours within the group contribute to individuals’ treatment outcomes.^16^

Clinicians often report a sense early on in treatment of whether a ‘group will go well or badly’^16^ (p161), but there is little research investigating such group effects within a chronic pain setting. One study within a chronic pain management programme showed a clustering of outcomes within groups, and suggested that levels of staff turnover adversely affected outcomes.^17^ Mixed modelling methods have been widely used to understand group context effects within other fields.^18^ However, to our knowledge, only two studies have used these methods to investigate group effects within CBT for chronic pain.^19,20^

Drawing on findings from psychotherapy^21^ and organisational,^22^ social,^23^ and educational^24^ psychology research, it has been suggested that certain features of group composition may impact individual pain management outcomes by influencing cohesion between group members and with group treatment goals.^16,19^ In one study, Wilson et al. and found that group size, homogeneity of sex and age and group compensation status were associated with individuals’ pain intensity and interference following group-based CBT for chronic pain.^19^ However, there were inconsistencies in findings between the two centres studied, making it difficult to draw firm conclusions and replication is needed. In another study, Wilson et al. explored whether the psychological composition of the group predicted individual outcomes, assuming from broader research that when groups exhibit adaptive behavioural norms (e.g. low group depression and fear-avoidance), assimilation to these group norms may be beneficial for the individual.^20^ However, their analyses using different data from the same group-based CBT as the earlier study found no consistent association between group scores on depression, pain catastrophising, self-efficacy, or fear-avoidance and individual outcomes.^20^

In addition to the group composition variables investigated by Wilson et al.^19,20^ there are numerous other potential demographic, pain and psychosocial variables to consider when exploring group composition effects on pain management outcomes. Research is needed to investigate a range of group composition variables within the same analysis to capture this complexity. In addition to age and sex, ethnicity may be a powerful cue for similarity which may influence group cohesion^25,26^ and thus the ethnic make-up of the group warrants investigation. At the individual level, there are mixed findings on the association between baseline pain intensity and interference and outcomes within contextual forms of CBT for chronic pain^12^; however, one RCT of online ACT suggests that individuals with higher levels of baseline pain intensity may respond better.^27^ Displays of pain and pain-related disability within the group may serve a particularly salient modelling function and, therefore, it seems plausible that group level pain intensity and interference might exert an impact on individual outcomes.

Research is needed to explore group composition on other psychosocial factors that are relevant for pain management. Within ACT, pain acceptance is a theoretically relevant treatment target which is associated with daily functioning at the individual level.^12,28,29^ Thus, group level acceptance may plausibly influence individual outcomes through normative modelling of adaptive responses within the group. Focussing more on interpersonal experiences, stigma is also associated with pain outcomes at the individual level.^30^ It is plausible that shared experiences of stigma could facilitate group cohesion through a sense of validation; alternately, overidentification with this group identity may impede treatment progress.^31^ Therefore, it may be useful to investigate group composition on experiences of stigma particularly given the interpersonal nature of this variable.

This study builds on previous research by using routinely collected data to investigate group composition in relation to individual outcomes in an ACT-based treatment for pain. The independent variables were group size and group composition in terms of gender, ethnicity and disability benefits status, and baseline pain intensity and interference, depression, pain acceptance and stigma. The independent group composition variables were selected to replicate and extend elements of the previous work of Wilson et al,^19,20^ as discussed. We hypothesised that these variables would be associated with the post-treatment individual level outcomes of pain intensity, pain interference, and depression. Given limited research in this area and inconsistent group context findings previously reported, we did not make specific hypotheses about the direction of effects.

## Methods

This was an observational study in which treatment and data collection were conducted as part of routine clinical practice. All participants were asked to complete self-report measures on the first and final day of treatment. Standardised, reliable and well-validated measures were used to minimise bias. The current study is a secondary analysis of routinely collected data. A manuscript describing portions of the current dataset has been published.^32^ The aim of the current analyses to explore group context effects is distinct from the previous publication which focussed on psychometric validation of a self-report measure not investigated here and individual level data only. Ethical approval for the research database was obtained from the Health Research Authority South Central – Oxford C Research Ethics Committee (17/SC/0537). The research was conducted in accordance with the Declaration of the World Medical Association. The study protocol was not pre-registered.

### Participants

Participants were a consecutive sample of adults (≥18 years old) with chronic pain (≥3 mon) who attended a residential, group-based, pain management programme at St Thomas' Hospital in London, UK, between January 2018 and October 2019. Participants were predominantly referred for assessment for the programme either by their pain consultant or general practitioner, with some referrals also made internally from other medical specialities within the same hospital. Participants were assessed by a physiotherapist and psychologist to determine the suitability of the programme. As judged during the multidisciplinary assessment, all participants were experiencing significant pain-related disability and/or emotional distress that warranted treatment. Participants were eligible for the programme if they were willing and able to participate in a group treatment in English. Due to the residential nature of the programme, participants were required to be able to perform basic self-care tasks so they could safely manage in the accommodation while attending.

Participants were excluded if they were seeking further invasive treatments for pain (e.g. surgery) or had other medical comorbidities requiring further investigation or treatment that would interfere with programme completion. Lastly, participants were excluded if they were experiencing mental health problems (severe depression with active suicidal ideation, active psychosis, etc.) expected to impair treatment engagement. All participants provided written informed consent for their data to be used for the purpose of research. From 583 patients attending the programme, 67 did not provide consent for their data to be used for research. Therefore, the study sample included 516 participants at the start of treatment.

### Treatment

Participants completed an intensive residential interdisciplinary pain management programme within a speciality pain service. Treatment was delivered 4 days per week (approximately 9:00 a.m. to 5:00 p.m. each day) over 3 weeks by a team of psychologists, physiotherapists, occupational therapists and nurses. The programme was based on ACT and thus aimed to improve functioning and quality of life through fostering psychological flexibility – the capacity to experience pain with openness, consciously focus on experiences in the present and consistently pursue valued activities.^33,34^

Consistent with the treatment model, sessions aimed to increase participants’ present-moment awareness and willingness to experience pain and related unwanted experiences when doing so enabled them to successfully engage in personally meaningful activities. Metaphors, mindfulness practice, experiential exercises and values-based goal setting were used. Brief education about the nature of persistent pain was provided. There were regular physical activity/movement sessions and opportunities to practice and build values-based activities during treatment. A summary of example programme content is shown in Appendix 1.

Treatment was delivered in a group setting. Groups were formed pragmatically based on participants’ position on the waiting list following multidisciplinary assessment; they were not purposefully composed based on demographic or other features. In general, the service aimed to have groups of 10 participants, which was deemed clinically optimal. Groups could be slightly smaller at times due to participants declining treatment prior to starting or scheduling difficulties. Some groups were at times ‘overfilled’ in attempts to mitigate against these potential issues, resulting in slightly larger groups. Outside of treatment sessions, participants were able to socialise during breaks and in the evenings, if they wished to do so.

### Self-report measures

At the start of treatment participants responded to questions about their gender, ethnicity, age, disability benefits status, and pain location and duration. The disability benefits status variable was not specific to individuals with compensable injuries following a motor vehicle or work-related accident as in the Wilson et al. study^19^ and captured any income a person received due to the impact of their illness on their ability to work or care for themselves. At pre- and post-treatment, participants also completed standardised self-report measures of pain intensity, pain interference, depression, pain acceptance and stigma.

#### Pain intensity

Average intensity of pain over the past week was self-rated by participants using a numerical rating scale from 0 (no pain) to 10 (extremely intense pain).

#### Pain interference

The seven-item Brief Pain Inventory (BPI) pain interference subscale^35^ was used to assess the impact of pain on daily functioning, encompassing general activity, mood, walking, work, relationships with other people, sleep and enjoyment of life. Participants rated items on an 11-point scale, ranging from 0 (does not interfere) to 10 (completely interferes). The pain interference score was computed by averaging the seven items; higher scores reflect a greater degree of interference. The pain interference subscale has been well-validated in patients with chronic pain.^36^ The pain interference subscale showed good internal consistency (current study pre-treatment α = 0.85).

#### Depression

The nine-item Patient Health Questionnaire (PHQ-9)^37^ was used to assess the severity of depression symptoms based on the DSM-IV diagnostic criteria for major depressive disorder. The items were rated on a four-point scale, ranging from 0 (not at all) to 3 (nearly every day). The total PHQ-9 score was attained by summing all items (maximum possible score: 27); higher scores indicate greater severity of depression symptoms over the past 2 weeks. The PHQ-9 has been well-validated in patients with chronic pain.^38^ The internal consistency of the PHQ-9 in the current study was good (α = 0.82).

#### Pain acceptance

The eight-item Chronic Pain Acceptance Questionnaire (CPAQ-8)^39^ was used to assess pain acceptance, which refers to willingness to engage in activities in the presence of pain and stopping unsuccessful efforts to avoid or control pain.^40^ Pain acceptance is a theoretically relevant treatment process at the individual level within ACT for chronic pain.^29,41^ As such, it is theoretically consistent to explore whether group level pain acceptance adds to the prediction of patient outcomes. CPAQ-8 items were rated on a seven-point scale ranging from 0 (never true) to 6 (always true). Total CPAQ-8 scores were produced by summing the items (maximum possible score: 48); higher scores reflect a greater degree of pain acceptance. The CPAQ-8 has demonstrated associations with key chronic pain outcomes.^39,42^ The CPAQ-8 showed acceptable internal consistency in the current sample (α = 0.77).

#### Stigma

The eight-item Stigma Scale for Chronic Illnesses (SSCI-8^43^) was used to assess enacted (four items) and internalised (four items) stigma related to having a chronic illness. Enacted stigma refers to negative attitudes expressed by others toward a person with a devalued characteristic; internalised stigma describes when a person comes to believe these negative self-referential attitudes.^43^ Previous research supports the psychometric properties of the SSCI-8 in a chronic pain sample; in individual level analyses, SSCI-8 total scores were uniquely associated with pain interference and depression symptoms when controlling for demographic factors, pain intensity and pain acceptance.^30^ Therefore, it appears relevant to assess the additional contribution of group level stigma to patient outcomes. SSCI-8 items were rated on a five-point scale, ranging from 1 (never) to 5 (always). A total score was obtained by summing all items (maximum possible score: 40); higher scores reflect a greater degree of stigma. The SSCI-8 showed good internal consistency in the current study (α = 0.88).

### Data analysis

Data analyses were conducted using SPSS version 26 (IBM, NY). Descriptive statistics were computed to characterise the sample. Linear mixed effects models were used to investigate relationships between pre-treatment individual and group level variables and post-treatment individual level outcomes, accounting for the clustering of participants by group. Post-treatment pain intensity, interference and depression symptoms were the dependent variables. Independent samples *t*-tests and Chi-squared tests were used to compare pre-treatment variables for participants with and without missing data at post-treatment. Pre-treatment variables with significant differences between participants with and without post-treatment data were included in the linear mixed models.^44^

Null models, which did not contain any predictors, were first computed to examine the partitioning of the variance in each dependent variable within and between groups. Intracluster correlations (ICCs) were computed from these models to determine the suitability of conducting multilevel analyses; higher ICCs indicate greater clustering of the observations within groups. ICCs of >0.05 have been suggested as a cut-off for pursuing multilevel analyses.^45^ For the linear mixed effects models, a variance components matrix and maximum likelihood estimation were used. Intercepts were modelled as random effects, while individual and group level predictors were modelled as fixed effects. The pre-treatment score of the outcome variable was included as a covariate.

Continuous individual level pre-treatment predictors were group mean centred, which reflects each participant’s deviation from their group’s mean on a given variable. Dichotomous variables (e.g. gender) were coded as 0 and 1 at the individual level; proportions for these variables were computed for each group (i.e. the percentage of women in a group from 0–100). Mean scores for each group on each pre-treatment predictor were also computed; these group variables were grand-mean centred.^23^ This approach to modelling the independent variables partitions the variance into within groups (i.e. individual) and between groups (i.e. group-level) components and enables investigation of contextual group effects.^18^

In the event that the ICC was still >0.05 after entering group size, group demographic proportions and group mean variables, group discrepancy scores on continuous variables were entered into the model to explore the potential impact of differences between group members on a given variable on individual outcomes.^18^ Group discrepancy scores were computed for each continuous predictor as the pre-treatment range on that variable for each group (e.g. participant with lowest depression score in group 1 subtracted from participant with the highest depression score in group 1).^18^ Statistical significance was set at *p* < .05.

The sample size was determined by the number of participants receiving treatment between January 2018 and October 2019. The number of groups rather than the number of participants is of primary importance when determining the sample size for linear mixed models with group level data; 50 groups has been recommended as sufficient.^46^ There was a substantial amount (21%) of missing data on participants’ self-reported age. To maximise power, the models were first run without individual and group level age variables. Sensitivity analyses were then conducted with individual and group level age variables in the models.

## Results

Analyses were conducted with 516 consenting participants in 59 groups at the start of treatment. Of these, 421 participants (81.6%) completed post-treatment questionnaires. Unfortunately, reasons for post-treatment questionnaire non-completion were not systematically recorded. Therefore, it is not possible to ascertain the exact proportion of missing data attributed to treatment non-completion versus purely missed questionnaires, although treatment non-completion will account for a portion of this.

Participants were mostly women (78.9%) with a mean age of 48 years (SD = 13.0). Participants were predominantly white (73.2%). More than half the sample (65.6%) was receiving disability benefits. The median pain duration was 10.0 years (range 0.9–61.2 years). The most common primary pain sites were spinal (41.2%) and widespread pain (18.7%). Treatment group size ranged from 7 to 13 patients (median = 10). Descriptive statistics for baseline group composition variables are shown in Table 1. Table 2 displays descriptive statistics for individual level outcome variables (pain intensity/interference and depression) at pre- and post-treatment.

**Table 1.** Descriptive statistics for pre-treatment group level predictor variables.

Group variable	Mean (SD)
Size	10.02 (1.11)
Gender (% women)	79% (13%)
Ethnicity (% white participants)	73% (17%)
Disability benefits (% yes)	65% (16%)
Pain intensity (group mean)	7.82 (0.59)
Pain interference (group mean)	7.83 (0.62)
Depression symptoms (group mean)	18.02 (2.03)
Pain acceptance (group mean)	16.33 (2.53)
Stigma (group mean)	23.49 (2.80)
Pain intensity (group range)	4.04 (1.34)
Pain interference (group range)	4.30 (1.53)
Depression symptoms (group range)	15.79 (4.14)
Pain acceptance (group range)	24.12 (6.45)
Stigma (group range)	21.36 (5.35)

Note: Group range is the group discrepancy score.

**Table 2.** Individual level outcome variables at pre- and post-treatment.

	Pre-treatment (mean, SD.)	*N*	Post-treatment (mean, SD)	*n*	*t*	*d*
Pain intensity	7.82 (1.48)	512	7.05 (1.78)	420	10.09*	0.47
Pain interference	7.83 (1.54)	515	6.41 (1.89)	417	18.13*	0.79
Depression	18.04 (5.46)	506	13.02 (6.41)	416	17.89*	0.82

*Paired-samples *t-*test, *p*=0.00.

*d=t*_paired_ $\sqrt{2\left(1-r_{12}/n\right)}$ ^60^; *d* = 0.20, small effect size; *d* = 0.50, medium effect; *d* = 0.80, large effect.^61^

There were no significant differences on any baseline individual level variable between participants with and without post-treatment data. No significant differences were observed on group size or baseline group mean variables for those with and without post-treatment data. Relative to participants who did not complete post-treatment questionnaires, participants who completed these were in groups that had a greater proportion of women (*t* (514) = 2.82, *p* = .005), white participants (*t* (135.25) = 2.63, *p* = .009), and participants receiving disability benefits (*t* (126.28) = 3.42, *p* = .001).

### Intracluster correlations

Post-treatment individual level pain intensity varied significantly between groups (Wald Z = 2.44, *p* = 0.02). The ICC (0.11) indicated that approximately 11% of the variance in post-treatment pain intensity occurred between groups. Similarly, pain interference at post-treatment significantly varied between groups (ICC = 0.09; Wald Z = 2.13, *p* = .03). However, post-treatment depression symptoms did not significantly vary between groups (ICC = 0.04; Wald Z = 1.19, *p* = 0.24). Therefore, multilevel models were conducted to examine the associations between pre-treatment group level predictors and individual post-treatment pain intensity and interference; a multilevel model for post-treatment depression was not warranted.

### Baseline group-level predictors of post-treatment pain intensity and interference

Model residuals were approximately normally distributed. For both models, a sensitivity analysis was conducted by removing outliers (standardised residuals greater than +/−2.5). Removing outliers did not substantially alter the models; therefore, outliers were retained. Scatterplots of standardised residuals against predicted values supported linearity and homoscedasticity.

#### Pain intensity

Table 3 displays the results of the multilevel model for post-treatment pain intensity. After introducing the pre-treatment individual and group level predictors (i.e. size, group demographics, and group mean scores), the between-groups variance in post-treatment pain intensity was no longer significant (Wald Z = 1.70, *p* = .09). However, the ICC (0.07) was greater than the cut-off of 0.05, suggesting there was further variability to be explained between groups. Therefore, group range predictors were introduced in the model for pain intensity. After entering group range predictors, the ICC was 0.04 (Wald Z = 1.13, *p* = 0.26), indicating that there was minimal further between-groups variability to be explained; however, significant within groups variance remained (Wald Z = 12.44, *p* = 0.00).

**Table 3.** Pre-treatment individual and group level predictors of post-treatment individual level pain intensity.

Predictors	Estimate (*B*)	SE	*df*	*t*	*p*
Individual-level variables					
Gender (woman)	−0.05	0.19	322.92	−0.27	0.79
Ethnicity (white)	−0.15	0.18	324.25	−0.83	0.41
Disability benefits (yes)	0.04	0.17	322.10	0.25	0.81
Pain intensity	0.57	0.06	323.74	8.89	0.00
Pain interference	0.02	0.07	318.44	0.24	0.81
Depression	0.04	0.02	324.34	1.95	0.05
Pain acceptance	0.01	0.01	318.25	0.93	0.36
Stigma	0.03	0.01	321.57	2.38	0.02
Group-level variables					
Size	−0.02	0.11	50.28	−0.14	0.89
Gender (% women)	0.00	0.01	66.09	0.54	0.59
Ethnicity (% white)	0.00	0.01	71.21	0.62	0.54
Disability benefits (% yes)	0.01	0.01	78.16	2.09	0.04
Pain intensity (group mean)	0.97	0.25	50.41	3.85	0.00
Pain interference (group mean)	−0.07	0.29	59.45	−0.24	0.81
Depression (group mean)	−0.05	0.06	58.61	−0.80	0.43
Pain acceptance (group mean)	−0.01	0.04	56.56	−0.31	0.76
Stigma (group mean)	0.03	0.04	63.68	0.95	0.35
Pain intensity (group range)	0.11	0.10	59.77	1.15	0.26
Pain interference (group range)	0.04	0.09	64.01	0.47	0.64
Depression (group range)	−0.05	0.03	51.40	−1.98	0.05
Pain acceptance (group range)	0.00	0.01	65.35	0.19	0.85
Stigma (group range)	0.03	0.02	52.16	1.63	0.11

Note: Group range is the group discrepancy score.

The model indicated that baseline group mean pain intensity (*B* = 0.97, *t* (50.41) = 3.85, *p* = .00) and group percentage of patients receiving disability benefits (*B* = 0.01, *t* (78.16) = 2.09, *p* = .04) were significantly positively associated with individuals’ pain intensity after treatment (Table 3). None of the other group level variables were significantly associated with pain intensity at post-treatment. Pre-treatment individual-level pain intensity and stigma were also significantly positively associated with post-treatment pain intensity. All other individual level pre-treatment variables were not significantly related to post-treatment pain intensity.

#### Pain interference

After introducing individual and group level predictors (i.e. size, group demographic proportions and group mean scores), the variability in post-treatment pain interference between groups was no longer significant (Wald Z = 0.80, *p* = .42). The ICC (0.03) was also below the cut-off (0.05), suggesting that investigation of further group-level predictors to explain the between-group variance was not warranted. Therefore, group range predictors were not added to the model. However, significant within groups variance remained (Wald Z = 12.37, *p* = .00).

Table 4 displays the results of the multilevel model for post-treatment pain interference. The model indicated that, of the group variables, only group mean pain intensity at pre-treatment significantly predicted individuals’ pain interference at post-treatment (*B* = 0.71, *t* (47.15) = 3.44, *p* = .001). Pre-treatment individual level pain intensity, pain interference and depression were also significantly positively associated with post-treatment pain interference. All other individual and group level pre-treatment variables were not significantly related to post-treatment pain intensity.

**Table 4.** Pre-treatment individual and group level predictors of post-treatment individual level pain interference.

Predictors	Estimate (*B*)	SE	*df*	*t*	*p*
Individual-level variables					
Gender (woman)	−0.18	0.19	323.89	−0.95	0.35
Ethnicity (white)	−0.23	0.18	323.78	−1.31	0.19
Disability benefits (yes)	0.25	0.17	323.95	1.45	0.15
Pain intensity	0.30	0.06	322.52	4.75	0.00
Pain interference	0.42	0.07	316.29	6.23	0.00
Depression	0.04	0.02	323.89	2.17	0.03
Pain acceptance	−0.01	0.01	318.42	−0.82	0.41
Stigma	0.01	0.01	320.95	0.77	0.44
Group-level variables					
Group size	0.06	0.08	47.11	0.75	0.46
Gender (% women)	0.00	0.01	64.40	0.56	0.58
Ethnicity (% white)	0.00	0.01	73.56	0.64	0.52
Disability benefits (% yes)	0.01	0.01	79.42	1.32	0.19
Pain intensity (group mean)	0.71	0.21	47.15	3.44	0.00
Pain interference (group mean)	0.15	0.23	48.73	0.66	0.51
Depression symptoms (group mean)	0.04	0.05	55.53	0.70	0.48
Pain acceptance (group mean)	−0.03	0.04	56.29	−0.70	0.49
Stigma (group mean)	0.04	0.03	61.89	1.06	0.29

#### Sensitivity analysis with age predictor variables

Individual level age (*B* = 0.00, *p* = .81) and group mean age (*B* = −0.03, *p* = 0.26) did not significantly predict post-treatment individual level pain intensity. Individual level age (*B* = 0.00, *p* = .84) and group mean age (*B* = −0.01, *p* = .76) did not significantly predict post-treatment individual level pain interference. Given that age variables were not significant in either model, these variables were removed from the models to maximise power.

## Discussion

This study investigated the association between group composition variables and individual outcomes following interdisciplinary ACT-based treatment for chronic pain. Group percentage of participants receiving disability benefits and group mean pain intensity at pre-treatment were significantly positively associated with individual level pain intensity at post-treatment. Group pre-treatment mean pain intensity was the only group variable that predicted post-treatment pain interference. The findings can guide future research into the processes by which groups exert an impact on outcomes in cognitive-behavioural treatments for pain.

The ICCs reported here indicate that 9 and 11% of the total variance in post-treatment pain intensity and interference occurred between groups. This is similar to a previous study which reported ICCs ranging from 0.02 to 0.13 for pain intensity, interference, and disability across two CBT group treatments.^19^ Another study of group-based CBT for pain reported ICCs ranging from 0.06 to 0.20 for pain catastrophising and a walking test, respectively.^17^ Our study extends those findings by also examining group clustering for depression (ICC = 0.04). Future research is needed to understand reasons for variability in the magnitude of group clustering across different variables and treatments.

We found that group percentage of participants receiving disability benefits was positively associated with individual level pain intensity at post-treatment. One study found that group percentage receiving benefits predicted pain-related disability and interference following CBT.^19^ Insofar as improvements during treatment may impact the receipt of benefits upon which group members are dependent for their basic subsistence, this threat could, understandably, impede treatment progress in the group. Additionally, injustice perceptions (i.e. appraisals of loss, unfairness and blame^47^) and anger are understandable responses to challenges associated with navigating the benefits system.^48^ At the individual level, perceptions of pain-related injustice and anger expression have been shown to negatively impact on the therapeutic alliance.^49^ Thus, group level injustice and anger may similarly disrupt clinicians’ working alliance with the group. In the context of a poor working alliance between group and clinicians, individual group members may be less willing to engage in treatment, particularly when it includes a focus on opening up to unwanted experiences.

There was a lack of association between individual outcomes and group size and composition in terms of age, gender, and ethnicity. In contrast, one study reported that outcomes were associated with group size and homogeneity of age, albeit in only one of the CBT treatments evaluated.^19^ Additionally, in that study, the proportion of women in the group was associated with pain interference/disability in two of six models.^19^ Taken together, the results from two studies (across three centres) indicate that group demographic composition effects may not generalise across contexts. Age, gender and ethnicity have been discussed as salient indicators for similarity, which may facilitate group cohesion.^16,19^ However, these variables in isolation may not produce a sense of similarity if group members differ on other potential indicators of similarity (e.g. socioeconomic status). It is important to consider that a sense of similarity is not necessarily determined by demographics. Rather, it is the outcome of categorising self and other group members as relatively similar (in contrast to relevant out-groups) on a contextually important domain.^50^ This dynamic process of group formation, the functions of group similarity and cohesion, and their impact on outcomes following group-based treatments for pain requires further direct investigation.

The two previous studies that investigated group demographic^19^ and psychosocial^20^ composition in relation to pain management outcomes did not investigate the impact of group level pain intensity. In the current study, group mean pain intensity at pre-treatment was positively associated with individual level pain intensity and interference at post-treatment, controlling for pre-treatment individual level pain intensity and interference. Research is needed to understand the behavioural processes by which individuals in groups with greater pain intensity experience poorer outcomes. Groups with higher pain intensity may collectively direct more attention toward pain and may exhibit greater modelling of unhelpful pain behaviours which exacerbates pain and reinforces restrictions in pain-related activities.^51,52^ A heightened focus on pain and pain reduction and increased modelling of pain behaviours at the group level may limit awareness of emotional and cognitive responses to pain and reduce engagement with exploring alternative behavioural responses. Surprisingly, however, pain acceptance – a key treatment process that reflects effective responding with pain^29^– at the group level did not predict outcomes.

The non-significant findings for group level psychosocial variables in this study is consistent with the general lack of associations between group level fear-avoidance variables and outcomes in one previous study.^20^ There are measurement challenges when investigating theoretically guided predictors of outcomes in cognitive-behavioural pain treatments. One challenge is accurately reflecting and reporting upon one’s behaviour, particularly at the start of treatment before different behavioural repertoires are explored. Additionally, measuring pain acceptance at one timepoint may not adequately reflect the context-specific nature of acceptance as conceptualised within functional contextualism.^41^ Therefore, session-by-session assessment of group level acceptance may be needed to capture its contextual nature and association with outcomes.

It is important to highlight that we constructed group level predictors based on available individual level data and we did not directly measure behavioural patterns between group members. Social psychological research has demonstrated the positive impact of shared social identity and group cohesion in terms of feelings of connectedness, a sense of meaning, purpose and worth, giving and receiving social support, increased sense of agency and improved health outcomes.^50,53–56^ Importantly, however, the function and impact of shared identity and group cohesion may differ across contexts.^23,31,57^ For example, sharing a sense of identification with other people who are living with and functionally limited by pain may create a sense of cohesion between group members. Dominance of this group identity, however, could negatively impact on adherence with treatment goals aimed at increasing functioning with pain. Direct measurement over the course of treatment of processes such as a positive bond between group members and groups’ coherence with the therapeutic model^57^ in relation to outcomes following group-based cognitive-behavioural pain treatments is needed to advance this area of research.

One strategy to directly investigate group context effects would be to examine interactions between group members within sessions in terms of therapeutic and counter therapeutic behaviours. It may also be useful to examine actions by clinicians delivering treatment that are associated with helpful and unhelpful group processes. Examining interactions between clinicians and group members may also be fruitful. A combination of session-by-session patient and clinician self-report measures, observer ratings of interactions within sessions, and qualitative assessments of group experiences are likely needed to understand the behavioural processes by which treatment groups exert helpful or unhelpful effects.

Although we found evidence of group clustering, the between-groups variance was relatively small and significant within-groups variance remained after including several individual level predictors. Among the individual level variables, there were relatively few significant predictors of outcome, which is consistent with research on individual level predictors of contextual cognitive-behavioural treatment outcomes for pain.^12,13^ Therefore, further research is needed to understand individual level behavioural processes that influence treatment outcomes. Rather than assessing individual level predictors at a single point in time before treatment, within-session assessment of theoretically guided treatment processes, such as psychological flexibility, may afford greater opportunity to understand behaviours early in treatment that promote successful outcomes.^12^

At present, there is limited and inconsistent evidence for composing cognitive-behavioural treatment groups for pain based on certain patient characteristics. Although this may seem discouraging, efforts to compose pain management groups in relation to certain characteristics would be challenging to implement in most settings. The sheer number of potential group composition variables represents a challenge for theory, research and clinical practice. The identification of key behavioural processes amongst group members and clinicians that explain the impact of a wide range of group composition features on treatment outcomes may be a more suitable approach to advance this work.

Considering the ACT-based approach to treatment used here, applying the psychological flexibility model^41^ to study interactions between group members and clinicians may be useful. For example, clinicians may become fused with thoughts about their own inability to help certain groups; in turn, this fusion may lead to interactions that are less open or coherent with the treatment model. Enhancing clinicians’ ongoing awareness of behavioural responses coordinated by their own thoughts and emotions toward certain groups may foster more effective responding.^58^ Research is needed to investigate the relations between clinician and group level psychological flexibility and, ultimately, outcomes for individual patients.

This study has several limitations. This was a secondary analysis of routinely collected data using indirect rather than directly assessed measures of group context, as mentioned. Although the number of groups was sufficient for multilevel analyses,^46^ our sample was smaller than two previous studies investigating group effects in cognitive-behavioural pain treatments.^17,19^ Similar to previous work,^19^ our analyses were based on available data from patients consenting for their data to be used for research. Group composition variables at the start of treatment were associated with completion of post-treatment questionnaires, which has not been previously reported to our knowledge. We addressed this in our models by including baseline group-level variables that were significantly different between patients with and without post-treatment data.^44^ Nonetheless, a small percentage of individuals who did not consent for their pre-treatment data to be used for research are not reflected in the analyses despite having potentially exerted an influence on the group during treatment. This remains an ethically challenging aspect of group-based research to address. The data were collected in the context of an intensive interdisciplinary ACT-based treatment with a sample comprised of primarily white women. Therefore, the generalisability of the results to other cognitive-behavioural group-based treatment formats for pain and more diverse groups is uncertain. The study protocol was not pre-registered. Protocols for future research in this area should be pre-registered, such as through the Open Science Framework.^59^

To conclude, this study suggests that there is clustering of individual level outcomes for pain intensity and interference within groups in the ACT-based treatment studied here. However, examining group variables using aggregated individual level data from a single timepoint at the start of treatment yielded few significant predictors of individual outcomes at the end of treatment. Direct assessment of individual and group level therapeutic and counter-therapeutic processes on a session-by-session basis is needed to advance understanding of who benefits from group treatments for pain and how. Improved understanding of why some groups contribute to better outcomes than others will enable the provision of more effective treatments. Future research that draws on findings and methods from group research more widely may help to optimise the effect of the group in pain management programs.

## Appendix 1 Overview of the Three-Week Residential Pain Management Programme

Programme clinical teams consist of Psychologist/s, Physiotherapist/s, Occupational Therapist/s and Pain Nurse/s.

Over the course of a programme, each discipline works with the group for the following number of sessions/hours:

Psychology - 22
Physiotherapy - 22
Occupational Therapy - 15
Pain Nursing - 8

Sessions focus on core psychological flexibility processes (acceptance, cognitive defusion, present moment awareness, values, committed action, self-as-context^33,34^). Some examples of typical programme content are outlined below*:

**Table:**

Discipline	Examples of session content for one day
Psychology	‘Passengers on the bus’ metaphor:
	Participants encouraged to connect with a scenario where they are drivers of a ‘bus’ through their life-journey. They focus on how internal experiences (e.g. thoughts, memories, emotions, sensations) are like ‘passengers’ who will try to influence the direction of the ‘bus’. Participants are supported to think about which directions they want to move towards in their own journey through life, and to feel in control of their decisions in the presence of difficult internal experiences (processes: defusion, acceptance, self-as-context, values, committed action)
Physiotherapy	Mindful movement:
	Participants are guided through gentle movement with a focus on noticing sensations, urges, thoughts, habits, and decision-making as they do this (processes: present moment awareness, acceptance, defusion)
Occupational Therapy	Goal-setting:
	Participants are supported to identify and work towards values-based goals, identifying potential barriers and sources of support (processes: values, committed action).
Pain Nursing	Pain medication education and reviews:
	Information is provided in relation to commonly prescribed pain medications, with focus on effectiveness and side-effects, and support is given to reduce dosages when this is important to the patient (processes: values, openness).
